# A Sparse Bayesian Technique to Learn the Frequency-Domain Active Regressors in OFDM Wireless Systems

**DOI:** 10.3390/s25144266

**Published:** 2025-07-09

**Authors:** Carlos Crespo-Cadenas, María José Madero-Ayora, Juan A. Becerra, Elías Marqués-Valderrama, Sergio Cruces

**Affiliations:** Departamento de Teoría de la Señal y Comunicaciones, Escuela Técnica Superior de Ingeniería, Universidad de Sevilla, Camino de los Descubrimientos, s/n, 41092 Seville, Spain; ccrespo@us.es (C.C.-C.); jabecerra@us.es (J.A.B.); emarques@us.es (E.M.-V.); sergio@us.es (S.C.)

**Keywords:** behavioral modeling, frequency domain, nonlinear model identification, OFDM, power amplifier, sparse Bayesian learning, Volterra series

## Abstract

Digital predistortion and nonlinear behavioral modeling of power amplifiers (PA) have been the subject of intensive research in the time domain (TD), in contrast with the limited number of works conducted in the frequency domain (FD). However, the adoption of orthogonal frequency division multiplexing (OFDM) as a prevalent modulation scheme in current wireless communication standards provides a promising avenue for employing an FD approach. In this work, a procedure to model nonlinear distortion in wireless OFDM systems in the frequency domain is demonstrated for general model structures based on a sparse Bayesian learning (SBL) algorithm to identify a reduced set of regressors capable of an efficient and accurate prediction. The FD-SBL algorithm is proposed to first identify the active FD regressors and estimate the coefficients of the PA model using a given symbol, and then, the coefficients are employed to predict the distortion of successive OFDM symbols. The performance of this proposed FD-SBL with a validation NMSE of −47 dB for a signal of 30 MHz bandwidth is comparable to −46.6 dB of the previously proposed implementation of the TD-SBL. In terms of execution time, the TD-SBL fails due to excessive processing time and numerical problems for a 100 MHz bandwidth signal, whereas the FD-SBL yields an adequate validation NMSE of −38.6 dB.

## 1. Introduction

Energy efficiency has become a primary design objective in modern wireless communications systems. In the RF front end, the power amplifier (PA) is the most energy-intensive device, and therefore, it is imperative to pay special attention to its efficient operation. The efficiency versus linearity trade-off of PAs is a well-known fact that the adoption of ever-wider communication signals has exacerbated. Moreover, as orthogonal frequency division multiplexing (OFDM) is a prevalent modulation scheme in today’s wireless standards, it has tightened design constraints due to its commonly large values of peak-to-average power ratio (PAPR). In this context, linearization techniques have evolved from feedforward RF linearization to the highly developed digital predistortion (DPD) architectures proposed nowadays [1].

DPD and behavioral modeling of PAs have been the subject of intensive research in the time domain (TD) based on an ample catalog of a priori pruned Volterra-based PA models to avoid an excessively large number of regressors and coefficients [2]. Examples of them are the memory polynomial (MP) model [3], the generalized memory polynomial (GMP) model [4], and the bivariate circuit knowledge Volterra (bi-CKV) model [5].

Sparse regression employing a posteriori information from real measurements emerges as a promising alternative to the a priori pruning of the Volterra series. Among these techniques, the combination of a greedy algorithm—i.e., orthogonal matching pursuit (OMP) [6] or doubly orthogonal matching pursuit (DOMP) [7]—for the selection of the active regressors and an information criterion for determining the best sparse model was proposed. Notice that, under the sparsity hypothesis of this approach, Volterra models with richer initial regressor sets yield better performance. Within the regressor’s pursuit context, a sparse Bayesian learning in a time-domain (TD-SBL) procedure was also proposed in [8] to identify the most likely PA model.

In contrast with the TD approaches, only a scarce number of contributions can be found in the literature devoted to the study of the PA nonlinear behavior and its compensation in the frequency domain (FD) [9,10,11]. An FD technique to evaluate nonlinear distortion was proposed in [9] for a DVB-T2 OFDM signal. The proposed scheme to model the PA distortion was based on two blocks: a dynamic filter dependent on the input spectrum and a memoryless nonlinear block. It is likely that a more complex model structure could further enhance the performance results presented in that paper. The concept of FD DPD for OFDM-based transmitters proposed in [10] allows for flexibly controlling the modeling performance in different parts of the spectrum. A relatively low-complexity MP model structure is considered for the PA in that paper to develop an FD learning approach. Again, a simple model structure is a limitation to achieve superior performance results.

In addition, for the scenario of a visible light communications (VLC) system where emphasis is placed just on the in-band content of the signal, an FD algorithm to compensate nonlinear distortion produced by light emitting diodes (LEDs) in OFDM signals was presented in [11]. The algorithm operated on the signal constellation and was based on applying Bayesian pursuit to obtain a sparse MP model matrix. It demonstrated the improvement in the error vector magnitude (EVM) performance under different configurations, thus positively impacting particular metrics for VLC systems. This study is constrained to the in-channel band in VLC systems and obtains no results for the entire signal including adjacent bands.

In this communication, a procedure to model wireless OFDM systems in the frequency domain is proposed using a more general model structure and not limited to the in-band content of the signal. The values of an M-QAM constellation are allocated to the subcarriers of the OFDM symbol, and after an inverse FT (Fourier transform), the OFDM signal is transmitted, likely distorted by the PA. The objective is to demonstrate a frequency-domain sparse Bayesian learning (FD-SBL) algorithm to identify a set of regressors capable of efficient nonlinear distortion prediction. The performance objective of this FD-SBL proposal must be at least equivalent to the aforementioned TD-SBL procedure. The FD-SBL algorithm is first proposed to identify the active FD regressors and estimate the coefficients of the PA model using the information from a given symbol; subsequently, these coefficients are employed to predict the distortion of successive OFDM symbols.

## 2. FD Bayesian Approach to Nonlinear Distortion

In this section, a novel frequency-domain sparse Bayesian treatment is proposed to solve the power amplifier (PA) modeling. The sparse Bayesian learning procedure in the conventional time domain (TD-SBL) was already detailed in [8]. To learn a model, the process is theoretically formulated to be implemented in complex-valued linear regression that maximizes the likelihood of the measured data, including regressor pursuit and identification, coefficient estimation, stopping criterion, and regressor deselection. Essentially, given an initial large set of candidate regressors, the result of this sparse Bayesian learning approach is the most likely model with a small fraction of active regressors. It is pertinent to indicate that OMP and DOMP procedures advanced in [6,7] are greedy algorithms enhanced with a statistical Bayesian Information Criterion to select the active regressors. Unlike those approaches, the TD-SBL proposal is a thoroughly Bayesian method.

Since these approaches were entirely compared in [8], where the TD-SBL presented adequate modeling performance and computational cost, here, we focus on the frequency-domain SBL and its comparative to TD-SBL.

Recalling TD-SBL, in the time domain, x and y are the vectors that hold *M* consecutive samples of the complex envelopes at the input and the output of the PA, respectively. Then, the measurement equation is as follows:

(1) y=Xh+ϵ,

where X is the conventional measurement matrix that holds *N* regressors, $h\in C^{N\times 1}$ is the vector of model coefficients, and ϵ stands for the unavoidable error that the model cannot capture.

The matrix X is shaped by the model structure, which holds one regressor in each of its columns, as follows:

(2) $$X=\left[\phi_{1}\;\phi_{2}\;\cdots \;\phi_{N}\right]\in C^{M\times N},$$

where $\phi_{r}$ is the *r*-th regressor of the model that generally consists of some kind of functions of the input complex signal x(k). For example, an MP model gives an initial set with fewer potential regressors than GMP or bi-CKV structures.

The unobserved PA output signal is a linear combination of regressors

(3) $$y_{d}=Xh=h_{1}\phi_{1}+h_{2}\phi_{2}+\cdots +h_{N}\phi_{N}.$$

Fourier-transforming this conventional model structure composed of TD regressors, the resulting FD regressors are now ${\vec{\Phi }}_{r}=F\left\{\phi_{r}\right\}$ so that the Fourier transform of $y_{d}$, denoted as the output signal spectrum ${\vec{Y}}_{d}=F\left\{y_{d}\right\}$, can be written as a linear combination of the transformed regressors, as

(4) $${\vec{Y}}_{d}=h_{1}{\vec{\Phi }}_{1}+h_{2}{\vec{\Phi }}_{2}+\cdots +h_{N}{\vec{\Phi }}_{N}=\Phi h,$$

where Φ is the FD measurement matrix shaped as $\Phi =[{\vec{\Phi }}_{1}\;{\vec{\Phi }}_{2}\cdots {\vec{\Phi }}_{N}]$. Therefore, for a given acquisition, we can write

(5) $$\vec{Y}=\Phi h+F\left\{\epsilon \right\},$$

where $\vec{Y}={\left[Y\left(0\right),\cdots ,Y\left(M-1\right)\right]}^{T}$ is a column vector with the acquired samples of the output spectrum and $F\left\{\epsilon \right\}$ is a column vector with the noise spectrum. In this case, the objective in (5) is to estimate the parameters h that best fit the observed full-spectrum vector $\vec{Y}$.

The parallelism of Equations (1) and (5) suggests that the TD-SBL can be directly adapted in this frequency-domain context to identify the active regressors and estimate the coefficients. In consequence, the regression vector h and the additive noise vector are assumed to be drawn from zero-mean random vectors, whose elements have respective variances $\alpha_{i}^{-1}$, i=1,⋯,N, and $\beta^{-1}$. Thus, the statistical approach [8] is directly adapted to the frequency domain by assuming a joint complex-valued Gaussian distribution for the coefficients h and the vector of measurements $\vec{Y}$. Accepting the independence of the samples, the likelihood of the FD dataset is given by

(6) $$p\left(\vec{Y}|h,\beta^{-1}\right)=\frac{1}{{\left(\pi \beta^{-1}\right)}^{M}}e^{-\beta ||\vec{Y}-\Phi h{\left|\right|}^{2}}.$$

In this Bayesian perspective, the parameters are constrained by defining an explicit prior probability distribution over them so that the likelihood function is complemented by a zero-mean Gaussian prior distribution over the coefficients h employing the auxiliary definition of the vector of hyperparameters $\left[\alpha_{1},\cdots ,\alpha_{N}\right]$. Please see [8] for more details.

Possibly, the more direct application of this FD-SBL method is to a segment of the signal equivalent to an OFDM symbol. The OFDM symbol to be transmitted is denoted as $\vec{X}={\left[X\left(0\right),X\left(1\right),\cdots ,X\left(N_{\mathrm{FFT}}-1\right)\right]}^{T}$, where X(m) is the M-QAM symbol at subcarrier *m* and $N_{\mathrm{FFT}}$ defines the total number of subcarriers. Therefore, $\vec{X}$ is an FD representation of the OFDM signal to be transmitted, with samples given by

(7) $$x\left(k\right)=\frac{1}{N_{\mathrm{FFT}}}\sum_{m=0}^{N_{\mathrm{FFT}}-1}X\left(m\right)e^{j2\pi km/N_{\mathrm{FFT}}},\;k=0,1,\cdots ,\;N_{\mathrm{FFT}}-1.$$

For convenience, in the following discussion, we use the sequence of indices given by $\left[-N_{\mathrm{FFT}}/2,N_{\mathrm{FFT}}/2-1\right]$, which involves swapping the left and right halves of the transform. Although an $N_{\mathrm{FFT}}$ -points transform is adequate for the linear regressors, a higher transform length is necessary to include the spectral content generated by the nonlinear regressors in the adjacent channels. For example, if a signal with a bandwidth *B* is described with a transform length of $N_{\mathrm{FFT}}$, third-order regressors generate spectral content in the adjacent channels within a 3B band, fifth-order regressors generate spectral content in a 5B band, and so on. To ensure accuracy, the $N_{\mathrm{FFT}}$ -points of the FT should be extended correspondingly. Next, we introduce the algorithmic steps of the SBL algorithm.

### 2.1. The FD Sparse Bayesian Pursuit

To estimate the coefficients h that best fit the observed spectrum $\vec{Y}$, we focus on (5). Recalling [8], the adaptation of the TD-SBP approach to the frequency domain leads to the following covariance matrix definition:

(8) $$C=\beta^{-1}I+\Phi A^{-1}\Phi^{H}$$

where $A=\mathrm{diag}\{\alpha_{1},\cdots ,\alpha_{N}\}$ is the prior precision matrix with a diagonal shaped by the hyperparameters $\alpha_{i}$. The maximization of the logarithm of the marginal likelihood (which gives the objective function) focuses on the dependence of C on a single hyperparameter $\alpha_{i}$ based on the following expression:

(9) $$C=\beta^{-1}I+\sum_{r\neq i}\alpha_{r}^{-1}{\vec{\Phi }}_{r}{\vec{\Phi }}_{r}^{H}+\alpha_{i}^{-1}{\vec{\Phi }}_{i}{\vec{\Phi }}_{i}^{H}=C_{-i}+\alpha_{i}^{-1}{\vec{\Phi }}_{i}{\vec{\Phi }}_{i}^{H},$$

where $C_{-i}$ is C without the contribution of the regressor *i* (and the dependence on $\alpha_{i}$ removed), and β is a parameter related to the noise present in the measurement. The sparsity factor $s_{i}$ and the quality factor $q_{i}$ in the FD are defined in this case as

(10) $$s_{i}={\vec{\Phi }}_{i}^{H}C_{-i}^{-1}{\vec{\Phi }}_{i}\;\mathrm{and}\;\;q_{i}={\vec{\Phi }}_{i}^{H}C_{-i}^{-1}\vec{Y}.$$

Notice that $s_{i}$ is a measure of the extent to which the spectrum regressor ${\vec{\Phi }}_{i}$ overlaps those regressors already present in the model, and $q_{i}$ gives a measure of how well ${\vec{\Phi }}_{i}$ increases the marginal likelihood by helping to explain the data. The objective function has a unique maximum with respect to $\alpha_{i}$ at

(11) $$\alpha_{i}=\frac{s_{i}^{2}}{|q_{i}{|}^{2}-s_{i}}\;\mathrm{if}\;|q_{i}{|}^{2}>s_{i}.$$

The pursuit is initiated with an empty active set of regressors; therefore, $C_{-i}=\beta^{-1}$ in (9), and the potential set is the full stock of the nonlinear regressors yielded by the selected behavioral model. Setting some sensible initialization, for example, $\beta^{-1}=10^{-5}$, the values of $s_{i}$ and $q_{i}$ are computed, and the potential regressor ${\vec{\Phi }}_{i}$ that maximizes the objective function

(12) $$\ell \left(\alpha_{i}\right)=\frac{|q_{i}{|}^{2}-s_{i}}{s_{i}}+\ln\frac{s_{i}}{|q_{i}{|}^{2}}$$

is incorporated to the set of active regressors.

This step not only selects the regressor with the greatest projection $|{\vec{\Phi }}_{i}^{H}\vec{Y}|$, as in other greedy pursuits, but also includes the additional condition $|{\vec{\Phi }}_{i}^{H}\vec{Y}{|}^{2}>\beta^{-1}$, so that those potential regressors with a projection below the experimental noise threshold are deleted by setting $\alpha_{i}=\infty$. The active set is increased by one regressor with a newly computed $\alpha_{i}$. Then the posterior covariance matrix Σ and the vector with the mean of the coefficients μ, which are scalars in this first iteration, are computed along with the updated values of $s_{i}$ and $q_{i}$ for all potential regressors. In each iteration, the regressor that maximizes the marginal likelihood is retrieved, and the posterior covariance and mean of the coefficients are updated. The final expressions are

(13) $$\Sigma ={\left(\beta \Phi^{H}\Phi +A\right)}^{-1}$$

and

(14) $$\mu ={\left(\Phi^{H}\Phi +\beta^{-1}A\right)}^{-1}\Phi^{H}\vec{Y},$$

where the a priori precision A is a diagonal matrix. During the regressor pursuit, many $\alpha_{i}$ tend toward infinity, meaning that these coefficients are peaked at zero, i.e., $h_{i}=0$, and the corresponding regressors are not included in the active set. The procedure result is a sparse active set that can be considered as the most likely reduced model.

Here, we can observe two relevant considerations. First, the coefficients are estimated with a sequential algorithm avoiding the expensive computation of the regressor matrix pseudoinverse. Second, the exposed regressors’ identification and coefficients’ estimation are feasible by implementing the FD-SBL employing only one OFDM symbol. In that case, it is conceivable that the validation performance can be enhanced with information provided by data from new OFDM symbols. A method to improve performance through optimal combination of prior and likelihood is discussed next.

### 2.2. Optimal Combining of Prior and Likelihood

The application of the FD-SBL technique to one OFDM symbol is initiated with the formal equation written as

(15) $${\vec{Y}}_{s}=\Phi_{s}h+F\left\{\epsilon_{s}\right\},$$

with *s* indicating the symbol under evaluation and the maximum likelihood covariance for this symbol is denoted as

(16) $$\Sigma_{s}={\left(\beta \Phi_{s}^{H}\Phi_{s}\right)}^{-1}.$$

After implementing the FD-SBL to the first OFDM symbol, s=1, the corresponding posterior covariance $\Sigma_{P1}$ and mean of the coefficients ${\hat{h}}_{P1}$ are given by Equations (13) and (14). Having said that, the precision of the identified sparse model can be improved by the optimal combination of prior and likelihood. With this optimal combination of information of *S* OFDM symbols, the enhanced posterior precision and mean of the coefficients can be rewritten as

(17) $$\Sigma_{PS}^{-1}=\sum_{s=1}^{S}\Sigma_{s}^{-1}+A$$

and

(18) $${\hat{h}}_{PS}=\Sigma_{PS}\left(\sum_{s=1}^{S}\Phi_{s}^{H}\beta \left({\vec{Y}}_{s}-\mu_{Y_{s}}\right)\right),$$

respectively. Observe that the posterior precision (17) increases as covariance information (16) from new symbols is incorporated, and the coefficients are accordingly updated by applying (18).

## 3. Experimental Results

### 3.1. Choice of the PA Model

Since conventional PA models (MP, GMP, bi-CKV) are defined in the time domain, there are no actual PA models available in the frequency domain. For that reason, the processing required by the technique in the FD necessitates the conversion of the TD regressors of the mentioned models into FD regressors. This conversion is easily achieved with a Fourier transform of the TD regressors, taking into account that the FFT size must be large enough to accommodate the band of the nonlinear regressors. On the other hand, the number of signal samples increases with large bandwidths. For example, in TD, the analysis of a 30 MHz bandwidth signal requires the management of regressors with about 300,000 samples in length, whereas a 100 MHz bandwidth signal involves regressors with a length of more than one million samples. Conversely, in FD, the complexity would be the same for 30 MHz and 100 MHz if the FFT size is unchanged.

In order to find the adequate basis functions, we considered conventional PA models. Researchers often consider low-complexity models, such as the MP structure, to be convenient. However, the implementation of pursuit methods, such as SBL, offers better results with more complex models that provide a larger number of regressors. Therefore, the GMP and the bi-CKV models, with superior performance and accredited efficiency, are preferable candidates. One simplified relationship of the bi-CKV model is [5]

(19) $$\begin{array}{c} y\left(k\right)=\overset{N}{\underset{n=1}{\sum^{\prime }}}\sum_{q_{2}=0}^{Q_{2}}h_{n}\left(q_{1},q_{2}\right)x\left(k-q_{1}\right){\left|x\left(k-q_{1}-q_{2}\right)\right|}^{n-1}+\\ \;\;\;\;\;\;\;\;\;\;\;\;\;\overset{N}{\underset{n=2}{\sum^{\prime \prime }}}\sum_{q_{2}=0}^{Q_{2}}h_{n}\left(q_{1},q_{2}\right)x\left(k-q_{1}\right){\left|x\left(k-q_{1}-q_{2}\right)\right|}^{n-1}. \end{array}$$

The prime (^′^) indicates that only odd *n* indices are included in the first sum, and the double prime (^″^) indicates that only even *n* indices are included in the second sum. Note that the GMP model can be considered a particular case of the bi-CKV model.

According to our experience, the bi-CKV model offers a clear improvement and has been our choice for implementing the PA input–output relationship. The model parameters, i.e., nonlinear order *N* and memory lengths $Q_{2}=\left[Q_{1}\;\;Q_{2}\right]$, are selected to minimize the modeling error for each experimental scenario. The procedure applies a training signal to identify the active regressors of the model, a small fraction of the full set of the model regressors in sparse systems, and estimate the coefficients. Here, we use the SBL method to compare performances in the time domain and frequency domain. In the case of FD-SBL, the training signal is one OFDM symbol, and the model precision is improved by combining several OFDM symbols. In the second stage, the model performance is validated by applying a new input signal generated with different OFDM symbols.

As the main figure of merit, we use the normalized mean squared error (NMSE) in the frequency domain. If $\vec{Y}$ represents the discrete spectrum of one OFDM symbol of the output signal acquired by the experimental setup and $\hat{Y}$ is the corresponding spectrum predicted by the model, then the metric of the model’s fidelity in the frequency domain is

(20) $$\mathrm{NMSE}[\mathrm{dB}]=10\log\left\{\frac{\sum_{m=0}^{M-1}{\left|Y\left(m\right)-\hat{Y}\left(m\right)\right|}^{2}}{\sum_{m=0}^{M-1}{\left|Y\left(m\right)\right|}^{2}}\right\}.$$

Although this metric is equivalent to the conventional NMSE utilized in the time domain if the indices *m* belong to the signal band and the adjacent channels’ bands, it introduces some particular features. If the indices *m* are restricted to the in-channel band, the NMSE is also a metric of the transmitted signal fidelity, related to the EVM. In the case of indices restricted to the adjacent channels, the NMSE is a metric of the out-of-band signal fidelity. Notice that these attributes are not present in the conventional time-domain NMSE. In this work, we concentrate on the NMSE figure computed with the entire spectrum of the output signal.

### 3.2. First Scenario with 30 MHz Bandwidth OFDM Signal

**Figure 1:**
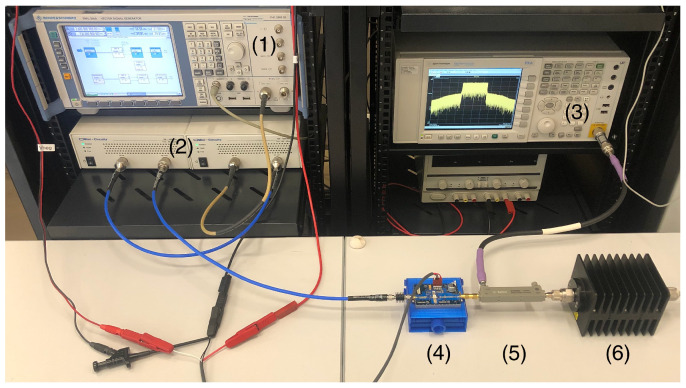
Photograph of the experimental setup composed of (1) a vector signal generator, (2) two cascaded preamplifiers, (3) a vector signal analyzer, (4) the PA under test, (5) a directional coupler, and (6) an attenuator terminated with a 50Ω load.

The experimental setup comprised a Rohde & Schwarz SMU200A signal generator, followed by two cascaded Minicircuits TVA-4W-422A+ preamplifiers working in a linear zone. The output signal was measured using a Keysight Technologies PXA-N9030A vector signal analyzer (VSA), with a power supply driving the PA. The PA under test was the evaluation board for the Cree CGH40010F GaN HEMT operating at a central carrier frequency of 3.6 GHz. The probe signal was an OFDM following the 5G-NR waveform format with a bandwidth of 30 MHz, a subcarrier spacing of 30 kHz, and an FFT size of 1024. The OFDM signal had a PAPR of approximately 11 dB and was generated with a sampling rate of 92.16 MHz. Subsequently, the signal analyzer acquired samples of the complex envelope at the output of the PA. The entire setup is presented in Figure 1.

In this experiment, the PA was operated with a moderate gain compression of 1.2 dB (Pout = +27.4 dBm), and modeling did not require a high nonlinear order or large memory depth. Under these conditions, a bi-CKV model with a nonlinear order N=9 and memory lengths $Q_{1}=10$ and $Q_{2}=2$ yielded an initial set of 1805 potential regressors. After applying the conventional TD-SBL pursuit, a set of fewer than 30 active regressors provided satisfactory performance with an identification NMSE of −50 dB. In the identification stage, two signals with TD lengths equivalent to 0.89% and 1.5% of the entire acquired signal length, respectively, were employed to compare performances. These signals span about one and two OFDM symbols, with 3281 samples and 5530 samples, respectively.

**Figure 2:**
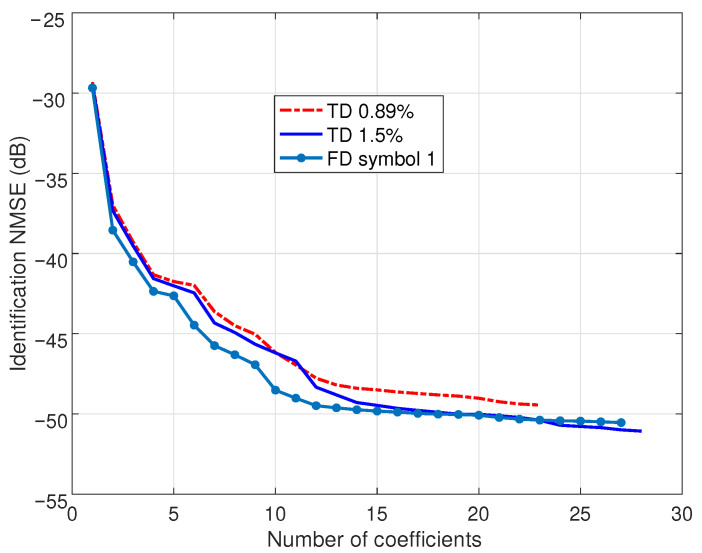
Performance of the TD-SBL and FD-SBL identification procedures. For the TD-SBL, two identification signals are employed with time-domain lengths equivalent to 0.89% and 1.5% of the entire acquired signal length. For the FD-SBL, the identification is performed by using frequency-domain data corresponding to one OFDM symbol.

Figure 2 shows the evolution of the identification NMSE in the course of the SBL procedures. For TD-SBL, the authors considered conventionally 1.5% of the total acquired signal (about two OFDM symbols). The identified model offers an NMSE of −51.1 dB with 28 regressors (see Table 1). Reducing the identification signal to 0.89% of the total signal (about one OFDM symbol), the model reduces to 23 regressors with a performance penalty, resulting in an NMSE of −49.4 dB. The present FD-SBL proposal was implemented with one OFDM symbol, and an FFT size increased to 4×1024=4096 points; the identified sparse model demonstrates a competitive NMSE of −50.5 dB with 27 regressors. The results of this figure demonstrate that the NMSE achieved by the FD-SBL method is superior to that of the TD-SBL with the 0.89% signal and comparable to the TD-SBL with the 1.5% signal.

**Figure 3:**
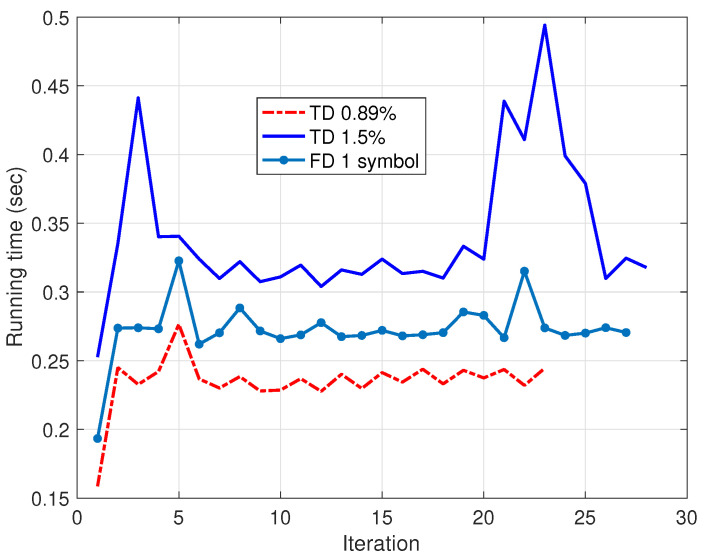
Execution time for the TD-SBL and FD-SBL identification procedures. For the TD-SBL, two identification signals are employed with time-domain lengths equivalent to 0.89% and 1.5% of the entire acquired signal length. For the FD-SBL, the identification is performed using frequency-domain data corresponding to one OFDM symbol.

On the other hand, the computational costs of the procedures under inspection are compared in Figure 3, employing the execution time in the same workstation. As expected, the execution time per iteration of the 1.5%-long TD signal is larger than that of the shortest one. However, the FD-SBL implemented with one OFDM symbol shows an intermediate execution time, allowing us to conclude that this novel proposal offers an economical method with a performance comparable to the time domain method.

**Figure 4:**
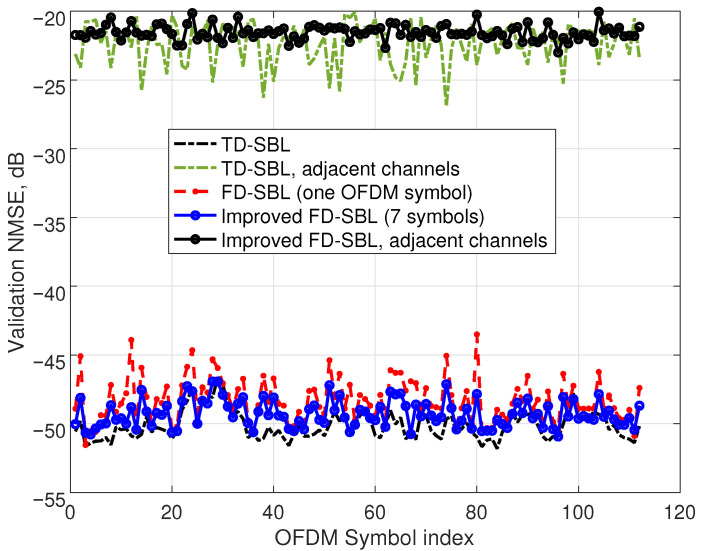
Validation NMSE for the 1-OFDM symbol FD-SBL identification and optimization with 7 OFDM symbols. For the TD-SBL, the identification is performed by using a time-domain length equivalent to 1.5% of the entire acquired signal length. Both the NMSE for the complete signal and the NMSE corresponding to just the adjacent channels are represented.

Finally, in this first experiment, we tested not only the accuracy in the identification stage but also the validation NMSE of the sparse models achieved by both procedures, TD-SBL and FD-SBL. Although the identification NMSE employing 1.5% of the acquired signal length with the standard TD-SBL model is about −51 dB, the computed validation NMSE employing the complete acquired signal degrades to a worst-case value of −46.6 dB, as shown with a dashed line in Figure 4. Notice that the NMSE corresponding to the adjacent channels has also been represented in this figure. Focusing on the complete validation signal with the FD-SBL model, the identification was performed with one OFDM symbol, and the validation NMSE was computed for all OFDM symbols (dashed line with marks), showing an NMSE deterioration with unfavorable results of about −44 dB. To ameliorate the results, the optimal combining technique incorporating the information from seven OFDM symbols was applied, demonstrating an improved validation NMSE of about −50 dB (plotted with a solid line with marks) and a satisfactory worst-case value of −47 dB, which compares favorably with the −46.6 dB of the TD-SBL technique.

In addition to these results, the NMSE corresponding to the adjacent channels has also been represented in the figure for the TD-SBL procedure (dashed line), yielding a worst-case value of −20 dB. The data computed by the FD-SBL are also plotted (solid line with circles), showing an equivalent value of −20 dB. Notice that these results provide information about the model precision concentrated in the adjacent channels, with poor accuracy values because the experimental noise is near the signal level in the adjacent channels.

### 3.3. Second Scenario with 100 MHz Bandwidth OFDM Signal

Once the performance of the FD-SBL procedure has been demonstrated with an experiment involving a 30 MHz bandwidth signal, the method is examined with the transceiver PVT360A from Rohde & Schwarz, followed by two cascaded Minicircuits TVA-4W-422A+ preamplifiers, to operate the PA under test in the saturation zone. The output signal was fed into PVT360A in the same way as in the previous setup. The PA under test was an RF Doherty PXAE261908NF from Wolfspeed, operating at a frequency of 2.6 GHz. The probe signal was a 5G-NR OFDM waveform with a bandwidth of 100 MHz, a subcarrier spacing of 60 kHz, and an FFT size of 2048. The probe signal was generated with a sampling rate of 614.4 MHz, which means an oversampling of 5, and was clipped to a PAPR of 9 dB.

The operation level of the PA was characterized by a gain compression of 2.7 dB, suggesting important expansion/compression and memory effects, which poses a more complicated scenario and a necessarily much richer structure for the PA model. For this task, the conventional TD-SBL technique was applied to the bi-CKV model with two structures. The first structure was a bi-CKV model similar to the previous scenario in order to achieve the most likely sparse set of regressors. For the second structure, we selected N=14 and longer memory depths with particular values for the different indices *n*. In this case, $Q_{1}$ spans values from 50 to 2 and $Q_{2}$ from 20 to 2, yielding an initial set of 5449 potential regressors. Once the sparse model was identified (with $N_{a}$ active regressors), its validation means processing the entire signal (1,228,800 samples) employing a 1,228,800 ×$N_{a}$ observation matrix and experiencing an excessive processing time. Additionally, it was not possible to identify the second structure due to numerical problems. From then on, we focused on the FD-SBL procedure.

**Figure 5:**
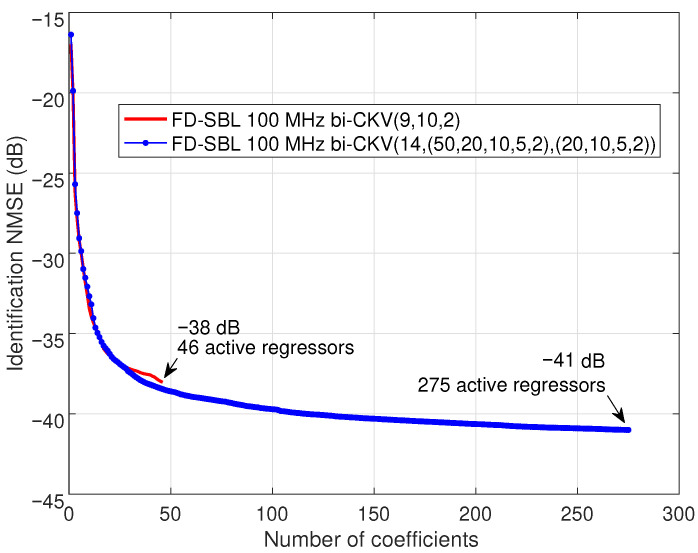
Performance of the FD-SBL procedure with a 100 MHz signal. Two bi-CKV models are considered. The model labeled as bi-CKV(9, 10, 2) has a maximum nonlinear order 9 and memory lengths $Q_{1}=10$ and $Q_{2}=2$. The model labeled as bi-CKV(14,(50,20,10,5,2),(20,10,5,2)) has a maximum nonlinear order 14 and memory depths with particular values for the different nonlinear-order terms, spanning from 50 to 2 for $Q_{1}$ and from 20 to 2 for $Q_{2}$.

The necessity of a more complex initial structure is observed in Figure 5. The evolution of the identification NMSE for the first model, plotted with a solid line and labeled as bi-CKV(9, 10, 2), demonstrates a most likely sparse model reaching an NMSE of −38 dB with 46 active regressors. On the other hand, the solid line with marks corresponding to the structure labeled as bi-CKV(14,(50,20,10,5,2),(20,10,5,2)), displays an NMSE of −41 dB with 215 active regressors for the most likely sparse model. It is remarkable that the FD-SBL technique can afford to manage the bandwidth increase to 100 MHz because the regressors’ length in FD depends on the FFT size and not on the number of samples of the signals.

**Figure 6:**
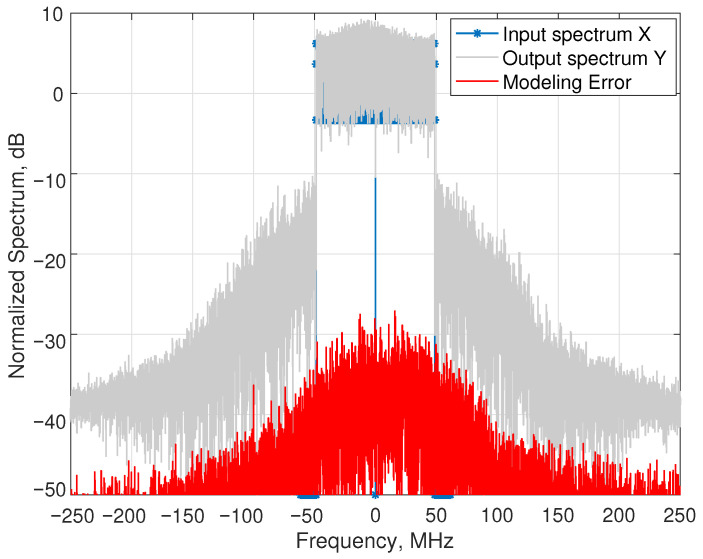
Input, output and prediction error spectra.

Once the sparse model has been identified by the FD-SBL method, the distorted output spectrum of a given symbol can be predicted from the input spectrum of that symbol. The M-QAM data are allocated to the 1620 active subcarriers of the OFDM symbol, and 2048 points of the input spectrum are generated by assigning zero values to the remaining subcarriers. The input spectrum shown in Figure 6 (solid line with marks) exhibits an effective bandwidth of 97.2 MHz. Since the oversampling was 5, the sampling rate was 5×2048×60kHz=614.4 MHz, allowing a reliable representation of a signal spectrum including the upper and lower adjacent channels. Then, an FFT size of 5 × 2048 = 10,240 points was necessary. Thus, the validation of the full signal is processed in a symbol-by-symbol basis involving a manageable 10,240 × 275 observation matrix.

The spectrum $\vec{Y}$ of the acquired output displayed in the same figure (gray line) shows the spectral regrowth generated by the nonlinear behavior of the PA. The spectrum has been scaled in order to be represented to match the input spectrum. To conclude this discussion, the error spectrum, computed as $\vec{Y}-\hat{Y}$, is represented with a solid line indicating the high accuracy of the method, about −40 dB below the in-channel signal level.

**Figure 7:**
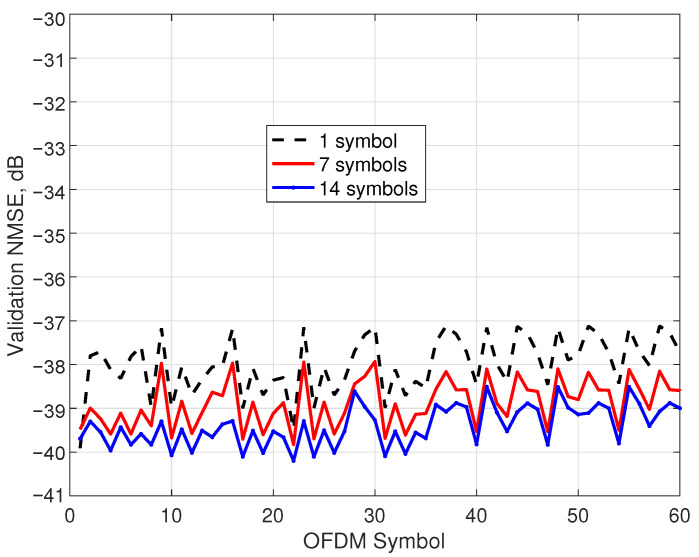
Validation NMSE of the FD-SBL identification without optimization (1 OFDM symbol) and with optimization (7 and 14 OFDM symbols).

Finally, in this experiment, the NMSE of the sparse model identified with the standard FD-SBL procedure was tested, and the models with improved performance were obtained with the aforementioned optimal combining technique. The validation NMSE for 60 OFDM symbols employing the model identified with the first OFDM symbol is represented in Figure 7 with a dashed line. The prediction reveals values of −37 dB, worsening by 3 dB the identification NMSE. The model was upgraded by incorporating information from 7 OFDM symbols (solid line) and 14 OFDM symbols (solid line with marks), with an evident improvement. In particular, the 14-symbols case demonstrates better validation NMSE values of about −40.2 dB and a reasonable worst value of −38.6 dB.

## 4. Conclusions

In this work, a novel FD-SBL algorithm has been proposed to identify sparse models in modern wireless systems with OFDM signals. The technique acts on the signal constellation information of the OFDM symbols and relies on a Bayesian pursuit in the frequency domain that corresponds to the standard SBL in the time domain. Unlike previous articles focusing on modeling and linearization with frequency domain techniques employing MP models, the proposed method can be applied to more general structures, like GMP and bi-CKV models. Each one of these models offers an ample set of candidate regressors from which the FD-SBL technique selects the active regressors that make up the sparse model. In this approach, the regressors have a length given by an FFT size adequate to accommodate nonlinear regressors and are processed in the frequency domain. The efficiency and execution time of the FD-SBL method are comparable to TD-SBL for a signal of 30 MHz bandwidth and clearly superior in the case of a signal of 100 MHz bandwidth, which presented numerical problems in our experiments. Recalling that, in OFDM transmitters, an essential block is the IFFT to transform the symbols to the time-domain signal, this technique can be integrated in the hardware to exploit the IFFT block by adding the linearization procedure. Therefore, the main potential application of the proposed FD-SBL algorithm is PA linearization by predistorting the OFDM symbols directly in the constellation domain, as it has been proved in other published articles for the singular case of the MP model [10,11].

## Figures and Tables

**Table 1:** Number and length of the regressors and NMSE of the sparse models determined with the TD-SBL and FD-SBL procedures. Labels of the TD identification signals are 0.89% and 1.5% for approximately one-symbol and two-symbol lengths, respectively.

TD-SBL (0.89%)	TD-SBL (1.5%)	FD-SBL (1 OFDM Symbol)
23 regressors	28 regressors	27 regressors
3281 samples	5530 samples	4096 spectral points
NMSE = −49.4 dB	NMSE = −51.1 dB	NMSE = −50.5 dB

## Data Availability

The data presented in this study are available on reasonable request from the corresponding author.

## Abbreviations

The following abbreviations are used in this manuscript:

OFDM	orthogonal frequency division multiplexing
M-QAM	M-ary quadrature amplitude modulation
FT	Fourier transform
FFT	fast Fourier transform
PA	power amplifier
TD	time domain
FD	frequency domain
MP	memory polynomial
GMP	generalized memory polynomial
bi-CKV	bivariate circuit knowledge Volterra
SBL	sparse Bayesian learning
NMSE	normalized mean squared error
EVM	error vector magnitude
